# Combined neurothrombectomy or thrombolysis with adjunctive delivery of 3K3A-activated protein C in acute ischemic stroke

**DOI:** 10.3389/fncel.2015.00344

**Published:** 2015-09-02

**Authors:** Arun Paul Amar, John H. Griffin, Berislav V. Zlokovic

**Affiliations:** ^1^Department of Neurosurgery, Keck School of Medicine of the University of Southern California, University of Southern CaliforniaLos Angeles, CA, USA; ^2^Department of Molecular and Experimental Medicine, Scripps Research InstituteLa Jolla, CA, USA; ^3^Department of Medicine, Division of Hematology/Oncology, University of California, San DiegoSan Diego, CA, USA; ^4^Zilkha Neurogenetic Institute, Keck School of Medicine of the University of Southern California, University of Southern CaliforniaLos Angeles, CA, USA

**Keywords:** activated protein C (APC), endovascular restorative neurosurgery, mechanical neurothrombectomy, neuroprotection, neurorestoration, stroke, thrombolysis

## Abstract

In the treatment of acute ischemic stroke (AIS), vessel recanalization correlates with improved functional status and reduced mortality. Mechanical neurothrombectomy achieves a higher likelihood of revascularization than intravenous thrombolysis (IVT), but there remains significant discrepancy between rates of recanalization and rates of favorable outcome. The poor neurological recovery among some stroke patients despite successful recanalization confirms the need for adjuvant therapy, such as pharmacological neuroprotection. Prior clinical trials of neuroprotectant drugs failed perhaps due to inability of the agent to reach the ischemic tissue beyond the occluded artery. A protocol that couples mechanical neurothrombectomy with concurrent delivery of a neuroprotectant overcomes this pitfall. Activated protein C (APC) exerts pleiotropic anti-inflammatory, anti-apoptotic, antithrombotic, cytoprotective, and neuroregenerative effects in stroke and appears a compelling candidate for this novel approach.

Stroke is the second leading cause of death worldwide and the number one cause of disability in the United States (Mozaffarian et al., 2015). Despite extensive research into the pathophysiology underlying acute ischemic stroke (AIS), intravenous tissue plasminogen activator (IV tPA) remains the only drug approved by the United States Food and Drug Administration (FDA) for its treatment. However, the time window for IV tPA eligibility is short, and there are contraindications for its use (Jauch et al., 2013). Furthermore, the (mis)perception of marginal utility, high risk of intracerebral bleeding, and/or high liability associated with its administration curtails the enthusiasm of many providers (SoRelle, 2013). As a result of these and other factors, only about 5% of AIS patients receive IV tPA (Jauch et al., 2013; Schwamm et al., 2013; Mozaffarian et al., 2015). Among those treated, rates of recanalization and good neurological outcome vary based on the site and size of the affected vessel, but can be as low as 5% for proximal occlusion of the internal carotid or basilar arteries (Bhatia et al., 2010). Clearly, a need exists for more effective reperfusion and neuroprotective strategies.

Mechanical neurothrombectomy has recently emerged as a promising approach to AIS therapy. The current generation of aspiration and stent retrieval devices achieves recanalization in the majority of patients (Table 1; Penumbra Pivotal Stroke Trial Investigators, 2009; Berkhemer et al., 2015; Nogueira et al., 2012; Saver et al., 2012, 2015a; Almekhlafi et al., 2014; Campbell et al., 2015; Goyal et al., 2015). Detailed analysis of adverse events and safety data confirms that neurothrombectomy procedures can be performed with minimal morbidity and mortality (Akins et al., 2014). Nonetheless, the likelihood of functional independence following neurothrombectomy (14–58%) remains poor compared with rates of recanalization (60–90%; Table 1).

**Table 1:** **Summary of major clinical trials of revascularization therapies**.

Trial name	Year published	Study type	Intra-arterial therapy	Adjunctive therapy	Recanalization (%)	mRS 0–2 (%)	sICH (%)	Mortality (%)	Comments
NINDS	1995	RCT	None	*IV tPA*	NR	39	6.4	17	Includes small vessel strokes and low NIHSS strokes
NINDS	1995	RCT	None	Placebo	NR	28	0.6	21	Includes small vessel strokes and low NIHSS strokes
PROACT I	1998	RCT	rpro-UK	IV heparin	57.7	30.8	15.4	26.9	Recanalization reported as TIMI 2 or more;
									outcome reported as mRS 0–1
PROACT I	1998	RCT	none	IV heparin	14.3	21.4	7.1	42.9	Recanalization reported as TIMI 2 or more;
									outcome reported as mRS 0–1
PROACT II	1999	RCT	rpro-UK	IV heparin	66	40	10	25	Recanalization reported as TIMI 2 or more
PROACT II	1999	RCT	none	IV heparin	18	25	2	27	Recanalization reported as TIMI 2 or more
IMS I	2004	Open label	tPA	IV tPA (low dose)	56	43	6.3	16	Recanalization reported as TIMI 2 or more
MERCI	2005	Single arm	Merci	None	46	27.7	7.8	43.5	Recanalization reported as TIMI 2 or more
IMS II	2007	Open label	tPA	IV tPA (low dose)	60	46	9.9	16	Recanalization reported as TIMI 2 or more
MULTI MERCI	2008	Single arm	Merci; +/− IA tPA	+/− IV tPA	68	36	9.8	34	Recanalization reported as TIMI 2 or more
Penumbra Pivotal	2009	Single arm	Penumbra	+/− IV tPA	81.6	25	11.2	32.8	Recanalization reported as TIMI 2 or more
SARIS	2009	Single arm	implanted stent	+/− IV tPA	100	60	5	25	Small series (*n* = 20)
SWIFT	2012	RCT	Solitaire	+/− IV tPA	61	58	2	17	Recanalization reported as TIMI 2 or more
SWIFT	2012	RCT	Merci	+/− IV tPA	24	33	11	34	Recanalization reported as TIMI 2 or more
Trevo 2	2012	RCT	Trevo	+/− IV tPA	86	40	7	33	Recanalization reported as TICI 2a or more
Trevo 2	2012	RCT	Merci	+/− IV tPA	60	21.8	9	24	Recanalization reported as TICI 2a or more
STAR	2013	Single arm	Solitaire	+/− IV tPA	79.2	57	1.5	6.9	
IMS III	2013	RCT	tPA and/or mechanical	IV tPA (variable dose)	38–44	40.8	6.2	19.1	Recanalization reported as TICI 2b or more
IMS III	2013	RCT	None	IV tPA	NR	38.7	8.9	21.6	
Synthesis	2013	RCT	tPA and/or mechanical	None	NR	42	6	8	
Synthesis	2013	RCT	None	IV tPA	NR	46	6	6	
MR Rescue	2013	RCT	IA tPA, Merci and/or Penumbra	+/− IV tPA	67	18.8	4.7	18.8	Recanalization reported as TICI 2a or more
MR Rescue	2013	RCT	None	+/− IV tPA	NR	20.4	3.7	24.1	
MR CLEAN	2014	RCT	Neurothrombectomy +/− IA tPA	+/− IV tPA	81.6	32.6	7.7	18.9	Recanalization reported as TICI 2a or more
MR CLEAN	2014	RCT	None	+/− IV tPA	NR	19.1	6.4	18.4	
ESCAPE	2015	RCT	Neurothrombectomy +/− IA tPA	+/− IV tPA	72.4	53	3.6	10.4	Recanalization reported as TICI 2b or more
ESCAPE	2015	RCT	None	+/− IV tPA	NR	29.3	2.7	19	
EXTEND IA	2015	RCT	Solitaire	IV tPA	100	71	0	9	Reperfusion assessed on CT imaging
EXTEND IA	2015	RCT	None	IV tPA	37	40	6	20	Reperfusion assessed on CT imaging
SWIFT PRIME	2015	RCT	Solitaire	IV tPA	88	61.2	1	9.2	Recanalization reported as TICI 2b or more
SWIFT PRIME	2015	RCT	None	IV tPA	NR	35.5	3.1	12.4	

*The disparities between rates of recanalization and good clinical outcome are highlighted, emphasizing the need for adjunctive therapy such as infusion of 3K3A-APC. Modified after Jadhav and Jovin (2013) and other sources. NR, not reported; RCT, Randomized Clinical Trial. See text for details and accompanying list for other abbreviations*.

The disparity between the rates of recanalization and rates of neurological recovery underscores the need for adjunctive therapy, such as pharmacological neuroprotection. Myriad preclinical studies and human trials with potential neuroprotective agents have been reported, yet none has proven unequivocally efficacious and none has achieved FDA approval (Ginsberg, 2009; Tymianski, 2013). Among the reasons cited for the failure of clinical trials in the face of encouraging animal data is the delayed time to administration, but even prehospital delivery of magnesium, given an average of just 45 min after symptom onset, failed to show benefit in a well-organized trial (Saver et al., 2015b). Another plausible explanation for the failed translation from bench to bedside is that the agent cannot reach the ischemic tissue due to lack of perfusion. When given systemically, neuroprotective agents must rely on collateral flow to ischemic tissue as they cannot traverse the occluded artery, but such collateral flow may be insufficient for adequate drug delivery. This provides impetus for a strategy coupling revascularization with the ancillary administration of a neuroprotective drug.

In this article, we review the foundation for an ongoing clinical trial coupling neurothrombectomy with adjunctive delivery of an activated protein C (APC) analog. APC confers pleiotropic benefits, such as stabilizing blood brain barrier (BBB) integrity, preventing thrombosis, enhancing fibrinolysis, promoting neuroprotection, attenuating inflammation, and facilitating neuroregeneration (Griffin et al., 2002, 2015; Zlokovic and Griffin, 2011). It represents a novel multiple-action multiple-target approach that ameliorates all facets of the pathogenic triad (consisting of vascular damage, neuronal injury, and neuroinflammation) that characterizes stroke and many other central nervous system (CNS) disorders (Zlokovic and Griffin, 2011). Since first report of the anti-inflammatory, cytoprotective, and antithrombotic properties of APC in stroke (Shibata et al., 2001), it has progressively fulfilled Stroke Therapy Academic Industry Roundtable (STAIR) criteria for drug development (Zlokovic and Griffin, 2011). The preclinical safety and pharmacokinetic profile of APC has been well characterized in mice and monkeys (Williams et al., 2012). A phase I safety study in normal human subjects has shown that high dose bolus regimens of modified APC are well-tolerated (Lyden et al., 2013), and a multicenter phase II dose-escalation clinical trial of intravenous administration for AIS (NCT02222714, NN104) is currently in progress (ZZ Biotech LLC, 2015).

## Limitations of Pharmacologic Thrombolysis

Data from intravenous thrombolysis (IVT) trials serve as the benchmark against which recanalization therapies such as neurothrombectomy are measured. Several small randomized trials of IV tPA suggested its safety efficacy in AIS (Mori et al., 1992; Haley et al., 1993), but large, randomized trials of another thrombolytic drug, intravenous streptokinase, were stopped early because of unacceptable rates of symptomatic intracranial hemorrhage (sICH; Donnan et al., 1995; Hommel et al., 1995).

The seminal National Institute of Neurological Disorders and Stroke (1995) trial reported favorable results that formed the basis for FDA approval of IV tPA in AIS. In this study, subjects were randomly assigned to receive either 0.9 mg/kg IV tPA (maximum 90 mg) given within 3 h of symptom onset or placebo. Due to the Phase 2 design of this pilot study, there were numerous exclusion criteria such as systolic blood pressure above 185 mm Hg or diastolic blood pressure above 110 mm Hg, prior stroke or head trauma within 3 months, major surgery within 14 days, history of intracranial bleed, anticoagulant use, platelet counts below 100,000 mm^3^, blood glucose concentration above 400 mg per deciliter, and others. The median National Institutes of Health Stroke Scale (NIHSS) score was 14 (range 1–37). There was a powerful and statistically significant benefit shown: at 3 months, the odds ratio for favorable outcome was 1.7 in the tPA group as compared with placebo. There was also a 12% absolute increase and 32% relative increase in the number of patients with minimal or no disability (Barthel Index 95–100) in the tPA group. Although there was no statistically significant difference between the group given tPA and that given placebo in the percentage of patients who showed a 4-point neurological improvement at 24 h (47 vs. 39% respectively, *p* = 0.06), using any other cut point yielded a highly significant benefit at 24 h (Haley et al., 1997). In addition, a long-term benefit 1 year later was observed for the tPA group using a global test statistic that represents a composite of other scales, including modified Rankin score (mRS). sICH within 36 h after stroke onset occurred in 6.4% of patients given tPA but only 0.6% of patients given placebo (*p* < 0.001). Mortality at 3 months was 17% in the tPA group and 21% in the placebo group (*p* = 0.30).

The benefit of IVT was confirmed in several subsequent, independent clinical trials (Hacke et al., 2008; Group et al., 2012). An ensuing meta-analysis of nine randomized trials confirms the robust benefit of IV tPA for AIS, with earlier administration associated with bigger proportional gain (Emberson et al., 2014). Among patients treated within 3 h, good outcome occurred in 259 (32.9%) of 787 patients given IV tPA vs. 176 (23.1%) of 762 who received control (OR 1.75, 95% CI 1.35–2.27). Treatment instituted after 3 h but before 4.5 h resulted in good outcome for 485 (35.3%) of 1375 patients vs. 432 (30.1%) of 1437 (OR 1.26, 95% CI 1.05–1.51). Delayed treatment beyond 4.5 h was not associated with statistically significant benefit.

In the NINDS trial, the size and location of the occluded vessel was not reported, as there was no time for vascular imaging. Stroke subtype (e.g., lacunar vs. large vessel) was determined using accepted clinical definitions. Similarly, there could be no data about recanalization. Subsequent studies have found that rates of recanalization with IV tPA alone can be as low as 5–10% for proximal large vessel occlusion (LVO), but much higher for more distal occlusions (Bhatia et al., 2010). Therefore, it is likely that the trials of IV tPA conducted without vessel imaging may have included patients with small vessel stroke and thus may overestimate any potential benefit to patients with LVO.

Due to these low rates of recanalization with LVO, various exclusion criteria, the short time window for benefit, the modest rates of good outcome, and other limitations of IV tPA, investigative efforts have focused on alternative revascularization strategies for LVO, including intra-arterial therapy (IAT). Such approaches include *in situ* delivery of thrombolytic drugs or other pharmacologic agents as well as neurothrombectomy. Currently, IAT is performed in patients who fail to recanalize after IV tPA or those who are ineligible for IV tPA on the basis of time or other exclusion criteria.

Local delivery of intra-arterial thrombolytics in animal models and small case series of human subjects suggested the safety and feasibility of this approach (Jadhav and Jovin, 2013), leading to subsequent randomized clinical trials (RCTs) such as the Prolyse in Acute Cerebral Thromboembolism (PROACT) I and II trials (del Zoppo et al., 1998; Furlan et al., 1999), which studied the intra-arterial delivery of recombinant pro-urokinase (rpro-UK) among patients with proximal middle cerebral artery occlusion (MCAOs: M1 or M2 segments) treated within 6 h of stroke onset. All patients also received adjunctive intravenous heparin, which likely contributed to rates of recanalization and/or reperfusion hemorrhage. Recanalization was assessed using the Thrombolysis in Myocardial Infarction (TIMI, 1985) score, which ranges from 0–3. Using an IAT dose of 6 mg rpro-UK and high vs. low doses of iv heparin, PROACT I proved superior rates of recanalization with thrombolytic (58%) than with control (15%; del Zoppo et al., 1998). Hemorrhagic transformation causing neurological deterioration within 24 h of treatment occurred in 15.4% of the rpro-UK group and 7.1% of the placebo group, but the difference was not statistically significant due to small sample size. Both recanalization and hemorrhage frequencies were influenced by heparin dose. Therefore, in an effort to increase recanalization while decreasing sICH, PROACT II used an IAT dose of 9 mg pro-UK and low dose IV heparin. This study also demonstrated superior rates of recanalization with thrombolytic (66%) than with control (18%; Furlan et al., 1999). Rates of functional independence (mRS 0–2) were also higher among the rpro-UK group (40%) than control (25%). Hemorrhagic transformation causing neurological deterioration within 24 h of treatment occurred in 10% of the rpro-UK group and 2% in the control group. Overall rates of hemorrhage, however, were as high as 68% at 10 days in the rpro-UK cohort, emphasizing that many hemorrhages within the infarcted territory do not produce incremental deficit. Ultimately, rpro-UK IAT was not pursued further for AIS.

Following on the promising results of PROACT, the IMS Study Investigators (2004) and IMS II Trial Investigators (2007) trials assessed a combined intravenous and intra-arterial approach to recanalization. In these prospective open label studies, reduced dose (0.6 mg/kg, 60 mg maximum) IV tPA was given within 3 h, followed by adjunctive IAT using up to 22 mg of tPA delivered at the site of occlusion. Compared with age- and severity-matched historical controls of IV tPA alone from the NINDS trial, the suggestion of improved outcomes with the combined IV and IA approach formed the rationale for IMS-III, a subsequent phase III RCT (Broderick et al., 2013).

## Mechanical Neurothrombectomy: Lessons Learned

In 2004, the Merci device (Stryker Neurovascular, Kalamazoo, MI, USA) became the first mechanical clot retriever to receive FDA clearance for AIS. The Merci system consists of a nitinol wire with a helical terminus that is deployed distal to the occlusion and then withdrawn proximally after engaging thrombus within its corkscrew structure. A balloon integrated into the guiding catheter is inflated during this process in order to arrest anterograde flow, thereby mitigating against the dislodgement of distal emboli. The Mechanical Embolus Removal in Cerebral Ischemia (MERCI) trial was a single arm, prospective, multicenter study large vessel stroke treated within 8 h of symptom onset who were ineligible for IV tPA (Smith et al., 2005). Recanalization (TIMI 2–3) was achieved in 46% of patients. Favorable outcome at 90 days (mRS 0–2) occurred in 27.7% of subjects overall, but there was a significant difference between those with recanalization (46%) and those without (10.4%). Similarly, overall mortality at 90 days was 43.5% overall but was better for those with recanalization (31.8%) than those without (54.2%). sICH was observed in 7.8%.

The Multi-MERCI trial was also a single arm, prospective, multicenter study of AIS patients with LVO treated within 8 h of stroke onset (Smith et al., 2008). However, some patients also received adjunctive IV tPA. In addition to the use of a newer generation Merci device, investigators could also perform salvage therapy with intra-arterial thrombolytic infusion. This approach increased the recanalization (TIMI 2–3) rate from 55% with the retriever alone to 68% overall. The rate of good outcome (mRS 0–2) at 90 days, however, remained disparately low at 36%. Mortality was 34% and sICH occurred in 9.8%.

In 2007, the Penumbra system (Penumbra Inc., Alameda, CA, USA) became the second FDA approved device for mechanical removal of thrombus in AIS. Unlike the Merci retriever, the Penumbra device does not require traversal of the clot and instead applies aspiration force to its proximal surface. Theoretically, this might reduce the incidence of distal emboli caused during the procedure and thus improve functional outcome. The Penumbra Pivotal Stroke Trial Investigators (2009) was a single-arm, prospective multicenter study of AIS patients with LVO who were either ineligible for or refractory to IV tPA. The device achieved an impressive revascularization rate of 81.6% using the thrombolysis in cerebral ischemia (TICI) scale, an analog to the TIMI rating (Higashida et al., 2003). However, only 25% had mRS 0–2 at 90 days, while another 33% died. The sICH rate was 11.2%.

Subsequent efforts at revascularization in AIS focused on stent technology, borrowing from the rich experience of these devices in the treatment of acute myocardial ischemia. Indeed, placement of implantable stents in AIS achieves high rates of recanalization, but it requires peri- and post-procedural antiplatelet therapy to prevent in-stent thrombosis, thus heightening the risk of reperfusion hemorrhage (Levy et al., 2009). In response, two new retrievable stents (“stentrievers”) were developed to exploit their efficacy in revascularization while eliminating the need to implant them *in situ*. As these devices are deployed within the clot, their struts engage the thrombus. When the stentriever is withdrawn, the entrapped clot is extracted from the body.

The first of these devices to gain FDA approval in 2012 was the Solitaire stent (Medtronic Inc., Minneapolis, MN, USA). In a study that directly compared its performance with that of the Merci device, the Solitaire yielded superior rates of recanalization (61% vs. 24%), mRS 0–2 (58% vs. 33%), sICH (2% vs. 11%), and mortality (17% vs. 38%; Saver et al., 2012). Detailed analysis of adverse events and safety data showed that minimal morbidity and mortality were attributable to the neurothrombectomy procedure itself (Akins et al., 2014). A subsequent single-arm registry confirmed these favorable results (Almekhlafi et al., 2014). The Trevo stentriever (Stryker Inc., Kalamazoo, MI, USA) became the fourth (and currently last) device to gain FDA approval for mechanical clot removal in AIS. In a study that directly compared its performance with that of the Merci device (Nogueira et al., 2012), the Trevo also yielded superior rates of recanalization (86% vs. 60%), mRS 0–2 (40% vs. 21.8%), and sICH (7% vs. 9%), but not mortality (33% vs. 24%, NS).

Although these single arm studies or randomized comparisons of various neurothrombectomy devices suggested progress compared with historical controls from IV thrombolysis trials, direct comparisons were lacking, and it remained uncertain whether or not the higher rates of recanalization achieved with IAT would translate into improved clinical outcome. Three subsequent RCTs failed to support the superiority of IAT over IV tPA alone (Broderick et al., 2013; Ciccone et al., 2013; Kidwell et al., 2013). However, all of them were subject to methodological flaws that call their validity into question.

The SYNTHESIS trial randomly assigned AIS patients less than 4.5 h from symptom onset to endovascular therapy (intra-arterial thrombolysis with tPA, mechanical clot disruption or retrieval, or combinations thereof) or standard dose IV tPA (Ciccone et al., 2013). The rates of good outcome (mRS 0–2) were comparable between IAT and IVT (42% vs. 46%) as were rate of sICH (6% vs. 6%) and mortality (8% vs. 6%), respectively. Of note, vessel imaging and confirmation of LVO pre-enrollment were not reported, and almost half of the patients had NIHSS <11, making LVO less likely in those subjects. Similarly, the locations of vessel occlusion and rates of LVO or recanalization were also not reported. Few IAT patients actually received neurothrombectomy, and the newer generation devices were rarely employed. Furthermore, IAT was instituted 1 h later (3.75 vs. 2.75 h) than IVT on average. Lastly, the withholding of IV tPA in the IAT group represents a departure from real world practice and likely contributed to lower rates of good clinical outcome in this group.

The IMS III trial randomly assigned eligible patients who received IV tPA within 3 h of symptom onset to receive additional IAT or IVT alone (Broderick et al., 2013). The trial was terminated for reasons of futility when a prespecified stopping rule was triggered, after it was demonstrated that rates of mRS 0–2 at 90 days were similar in the IAT and IVT groups (40.8% vs. 38.7%, respectively). Rates of sICH (6.2% vs. 5.9%) and mortality at 90 days (19.1% vs. 21.6%) were also similar. As with the SYNTHESIS trial, however, IMS III suffers from several criticisms that call into question its relevance to contemporary practice. First of all, vessel imaging and confirmation of LVO were not required. In the IAT group, many received the lower dose of IV tPA as per the prior IMS trials (0.6 mg/kg, 60 mg max) while only some received full dose (0.9 mg/kg, 90 mg maximum). Since the trial was conducted over 6 years, the nature of IAT varied according to available technology but mostly consisted of intra-arterial thrombolysis (80%) followed by use of earlier generation neurothrombectomy devices such as Merci (28%) or Penumbra (16%), while only 1.5% of patients were treated with stentrievers. Thus, rates of reperfusion according to the standard metric of TICI 2b-3, ranged from 38–44% according to location. Lastly, as with SYNTHESIS, there was significant delay in the initiation of IAT of up to more than 1 h.

In retrospective analysis of the prior trials, outcomes were generally worse among patients with established infarction on initial imaging. The Mechanical Retrieval and Recanalization of Stroke Clots Using Embolectomy (MR RESCUE) sought to assess the benefit of IAT in the presence of a penumbra of vulnerable tissue surrounding a small core infarct, based on the premise that salvage of this area through reperfusion might improve clinical outcome (Kidwell et al., 2013). The trial randomly assigned patients within 8 h of symptom onset to receive neurothrombectomy (with Merci or Penumbra) or standard care. Revascularization was achieved in 67% of the IAT group. However, at 3 months, rates of good outcome among the IAT and medical therapy groups were similarly poor (18.8% vs. 20.4% overall) whether or not a penumbral pattern was present (21% vs. 26%, 17% vs. 10%, respectively), as were those for sICH and death (Table 1). Criticisms of this trial include the long delay to initiation of IAT and the use of first generation neurothrombectomy devices.

Seeking to redress many of the problems of the trials showing no benefit of IAT over IVT alone, 4 RCTs were recently conducted, all proving the superiority of IAT when LVO is confirmed, newer generation devices are employed, appropriate adjunctive therapy is administered, and attention is focused on timely intervention (Berkhemer et al., 2015; Campbell et al., 2015; Goyal et al., 2015; Saver et al., 2015a).

The Multicenter Randomized Clinical Trial of Endovascular Treatment for AIS in the Netherlands (MR CLEAN) trial randomly assigned patients to intra-arterial treatment plus usual care or usual care alone (Berkhemer et al., 2015). All patients had proximal arterial occlusion in the anterior circulation confirmed by vessel imaging. Prior to enrollment, 89% of patients received IV tPA. Retrievable stents were used in 81.5% of patients assigned to interventional treatment, and TICI 2a or greater recanalization was achieved in 81.6% of patients. The rate of functional independence (mRS 0–2) with intervention (32.6%) was greater than that of control (19.1%) but was still comparatively low, reinforcing the unmet needs in AIS care. There were no significant differences in the occurrence of symptomatic intracerebral hemorrhage (7.7% vs. 6.4%) or 30-day morality (18.4% vs. 18.9%) in the interventional and control arms, respectively.

The Endovascular Treatment for Small Core and Anterior Circulation Proximal Occlusion with Emphasis on Minimizing CT to Recanalization Times (ESCAPE) trial compared IAT plus standard care vs. standard care alone in AIS patients with a small infarct core, proximal intracranial arterial occlusion, and moderate-to-good collateral circulation (Goyal et al., 2015). Importantly, patients up to 12 h after symptom onset were included. The study was terminated early due to the favorable rates of good outcome (mRS 0–2) in the interventional group (53%) vs. control (29.3%). Recanalization (TICI 2b or greater) occurred in 72.4%. sICH occurred in 3.6% of the intervention group and 2.7% of control, and mortality was reduced in the interventional group compared with control (10.4%, vs. 19.0%, respectively).

The Extending the Time for Thrombolysis in Emergency Neurological Deficits—Intra-Arterial (EXTEND-IA) trial studied AIS patients in whom CT perfusion confirmed the presence of salvageable brain and small infarct core (Campbell et al., 2015). All received standard dose IV tPA. Half were randomized to IVT alone while the other half was randomized to IAT using the Solitaire device. Reperfusion (assessed on CT imaging and thus on a different scale than TICI) was 100% in the Solitaire arm compared with 37% in the tPA-only group. Good functional outcome of mRS 0–2 was also more likely in the IAT group (71%) than the control (40%). Rates of sICH (0% vs. 6%) and mortality (9% vs. 20%) were also better for the IAT group.

The Solitaire With the Intention For Thrombectomy as Primary Endovascular Treatment (SWIFT PRIME) Trial is the most recently completed RCT of IAT vs. IVT alone for AIS (Saver et al., 2015a). All patients received full dose IV tPA. Using the Solitaire device, the IAT arm demonstrated an 88% rate of recanalization. Good functional outcome (mRS 0–2) occurred in 60.2% of the interventional group compared with 35.5% in the IV tPA arm. The rate of sICH (1%) in the Solitaire group was exceedingly low.

Collectively, these studies confirm that the newest generation of neurothrombectomy devices can achieve recanalization in the vast majority of patients. Even when neurothrombectomy is performed expeditiously among AIS patients with small infarct cores, however, the rate of good clinical outcomes is comparatively poor (Table 1). In real world settings outside the idealized circumstances of a clinical trial, clinical outcomes are likely to be even worse, thus reinforcing the need for adjunctive neuroprotective therapy, such as APC infusion.

## Overview of Activated Protein C

APC is an endogenous serine protease that favorably regulates multiple pathways within different cell types comprising the neurovascular unit, including neurons, vascular cells (endothelium, pericytes, and vascular smooth muscle cells), and glia (astrocytes, microglia, and oligodendroglia), all of which contribute to disease initiation and/or progression in AIS (Zlokovic and Griffin, 2011). APC is generated as part of the physiologic protective response to cerebral ischemia (Griffin et al., 2002). Further evidence of the importance of APC in stroke comes from prospective observational data suggesting that circulating levels of its zymogen precursor, protein C, are inversely related to the incidence of ischemic stroke (Folsom et al., 1999).

Protein C is a plasma zymogen that is synthesized in the liver and proteolytically activated by thrombin when bound to the endothelial protein C receptor (EPCR). The EPCR mediates many subsequent actions of APC (Domotor et al., 2003; Zlokovic and Griffin, 2011; Griffin et al., 2015) including transport across the BBB into the extravascular tissue (Deane et al., 2009) and activation of several cell signaling pathways, which ultimately affects the expression of hundreds of proteins (Griffin et al., 2015).

Conversely, dissociation of APC from EPCR allows its interaction with circulating clotting factors. By cleaving activated cofactors Va and VIIIa to yield the inactivated factors Vi and VIIIi, APC exerts potent antithrombotic effects. Replacement of a cluster of positively charged residues with neutral amino acids on the top surface of the APC protease domain near the C-terminus generates variants with reduced anticoagulant activity, but preserves the cell-signaling actions mediated by its N-terminus. For instance, substitution of three consecutive lysine residues 191–193 by alanine (3K3A-APC) causes loss of >92% of anticoagulant activity in human plasma and in monkeys compared with wild type APC (Zlokovic and Griffin, 2011; Williams et al., 2012). Such analogs are likely to prove favorable for therapeutic application in conditions where the cytoprotective properties are maintained, while the risk of serious bleeding is diminished (Williams et al., 2012).

**Figure 1:**
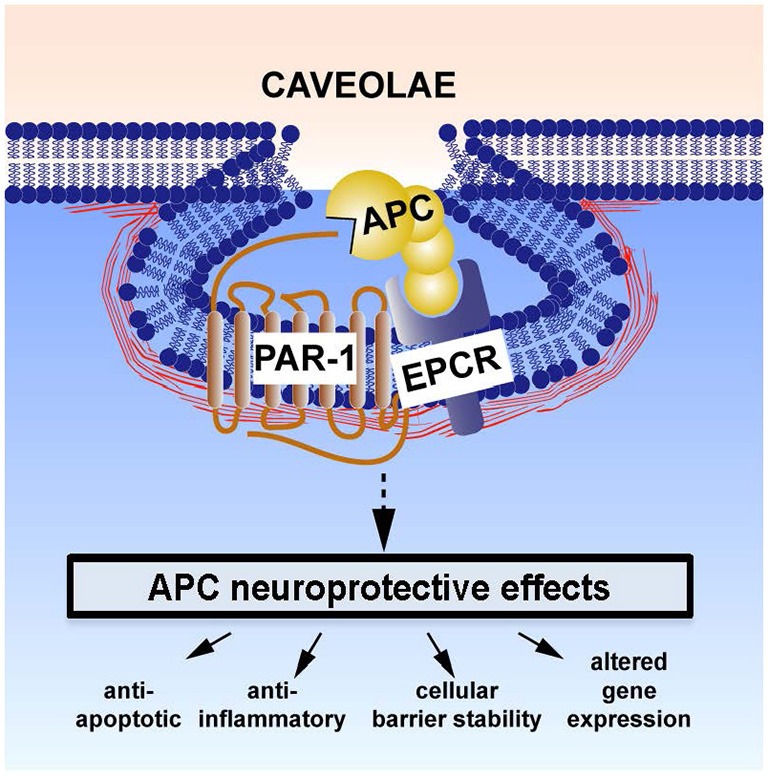
**Neuroprotective direct effects of APC on cells involves receptors endothelial protein C receptor (EPCR) and PAR-1**. The cellular receptors EPCR and PAR1 are required for APC’s beneficial effects on many types of brain cells. These activities include APC-mediated anti-apoptotic activities, anti-inflammatory activities, protection of endothelial barrier functions, and alterations of gene expression profiles. One or more of these activities plus other yet to be defined signaling actions are required for APC’s multiple neuroprotective activities (Zlokovic and Griffin, 2011). This paradigm in which EPCR-bound APC activates PAR-1 to initiate biased signaling (Griffin et al., 2015) is supported by many *in vitro* and *in vivo* data (Zlokovic and Griffin, 2011; Griffin et al., 2015). Localization of APC signaling in the caveolin-1 rich microdomains (caveolae) may help differentiate mechanisms for cytoprotective APC signaling vs. proinflammatory thrombin signaling (Zlokovic and Griffin, 2011; Griffin et al., 2015). Additional mechanisms for APC effects on cells may also involve other receptors, such as PAR-3, sphingosine-1-phosphate receptor 1, apolipoprotein E Receptor 2, and/or Mac1 (CD11b/CD18). For example, the beneficial actions of APC or 3K3A-APC on middle cerebral artery occlusion (MCAO) injury required PAR-1, EPCR, and PAR-3 (Cheng et al., 2003, 2006; Guo et al., 2004, 2013; Thiyagarajan et al., 2008; Gorbacheva et al., 2010; Petraglia et al., 2010; Zlokovic and Griffin, 2011) and stimulation of development of neurons within human neuroprogenitor cell populations required PAR-1, PAR-3, and sphingosine-1-phosphate receptor 1 (Guo et al., 2013).

EPCR-bound APC activates a family of G-protein coupled protease-activated receptors (PARs), including PAR1 (Domotor et al., 2003). The latter can also be activated by thrombin, but each ligand triggers divergent intracellular signaling cascades (biased agonism; Griffin et al., 2015). Whereas thrombin-mediated activation of PAR 1 leads to disruption of the BBB, vascular leakage, neurotoxicity, apoptosis, and neuroinflammation, APC-mediated activation of PAR1 produces the opposite effects. APC-induced biased signaling following PAR-1 activation is required for neuroprotective actions of APC (see Figure 1). These multiple downstream effects of APC have been reviewed elsewhere (Griffin et al., 2002, 2015; Zlokovic and Griffin, 2011) and are summarized below.

### BBB Preservation

The vasculoprotective effects of APC include PAR1-mediated activation of sphingosine kinase-1 which leads to activation of Rac1, a member of the Rho family of GTPases, and the rearrangement of the endothelial cytoskeleton. By stabilizing BBB integrity and attenuating post-ischemic BBB breakdown, APC prevents secondary neuronal injury due to the accumulation of blood-derived neurotoxic and vasculotoxic molecules such as fibrin, hemosiderin, thrombin, and plasmin (Zlokovic and Griffin, 2011).

APC also downregulates nuclear translocation of nuclear factor kappa B (NF-κB), thus blocking NF-κB dependent transcriptional activation of matrix metalloproteinase-9 (MMP-9). This molecule and other proteinases are responsible for post-ischemic degradation of the basement membrane proteins of the BBB, leading to intracerebral hemorrhage, an effect that is potentiated by tPA. In murine ischemic stroke models of MCAO, APC reduced tPA-induced intracerebral hemorrhage through its actions on PAR1 and reduced MMP9 expression (Cheng et al., 2006).

Additionally, APC blocks apoptosis of endothelial cells by inhibiting mitochondria-mediated caspase-9-dependent pathways, upregulating anti-apoptotic genes, suppressing p53 pro-apoptotic pathways, and other mechanisms (Cheng et al., 2003). Lastly, APC stimulates angiogenesis from endothelial cells in both *in vivo* and *in vitro* models, leading to the formation of new capillaries that can provide the nourishment for an environment conducive to neuroregeneration (Zlokovic and Griffin, 2011).

### Suppression of Neuroinflammation

APC inhibits transport of neutrophils and monocytes across the BBB, thereby blocking early post-ischemic infiltration of the brain by leukocytes. It also suppresses microglia activation. These effects are mediated by suppressing NF-κB dependent expression of proinflammatory cytokines such as tumor necrosis factor–α, interleukins, and vascular adhesion molecules (Zlokovic and Griffin, 2011).

### Neuroprotection

APC and its analogs cross the BBB via EPCR-dependent transport to reach neuronal targets and exert direct neuronal protection (Deane et al., 2009), as demonstrated in murine N-Methyl-D-aspartate (NMDA) excitotoxic injury models *in vivo* and in cultured neurons *in vitro* (Guo et al., 2004; Gorbacheva et al., 2010). Furthermore, as it does in endothelial cells, APC inhibits the intrinsic, caspase-9 dependent and p-53 mediated proapoptotic pathways and upregulates anti-apoptotic genes in neurons, too (Guo et al., 2004). Importantly, the therapeutic window of APC for stroke is much wider than that for tPA. Preclinical models demonstrate that APC is neuroprotective even when first administered 12 h after permanent MCAO or 24 h after transient MCAO (Guo et al., 2004; Wang et al., 2009). One key element for APC’s neuroprotection is its ability to signal activation of the Akt-survival pathways.

### Neuroregeneration

In addition to its neuroprotective effects, APC has been shown to promote post-ischemic neurogenesis in the mouse brain and in human embryo-derived neuroprogenitor cell cultures where activation of the Akt pathway is required (Thiyagarajan et al., 2008; Guo et al., 2013). These effects involve increased proliferation of neuronal progenitor cells of the subventricular zone and increased migration of neuroblasts from this area towards the ischemic border. Similar effects on neurogenesis were shown when APC was used in preclinical models of traumatic brain injury (Petraglia et al., 2010).

## Rationale for Combining Neurothrombectomy and Adjunctive APC

Untreated cerebral thrombosis is associated with high rates of morbidity and mortality. For instance, among patients with persistent proximal vessel in the anterior circulation, up to 80% die within 90 days of stroke onset or fail to regain functional independence (Goyal et al., 2015). Timely restoration of blood flow to the ischemic territory improves clinical outcome by salvaging the hypoperfused tissue at risk of infarction. Indeed, meta-analysis of several studies confirms the strong correlation between recanalization and outcome in AIS; the odds ratio of functional independence or death for those with recanalization compared to those without is 4.43 and 0.24, respectively (Rha and Saver, 2007). Similarly, for every 30 min delay in reperfusion, the likelihood of favorable outcome decreases by 10% (Khatri et al., 2009), as it is estimated that for each minute during acute stroke, 1.9 million neurons, 14 billion synapses, and 12 km (7.5 miles) of myelinated fibers are destroyed (Saver, 2006).

However, the premise that underlies this notion, the “recanalization hypothesis”, has been repeatedly contested (von Kummer et al., 1995; Rha and Saver, 2007). Analysis of the reasons behind this challenge reinforces the benefit of a strategy combining neurothrombectomy with adjunctive delivery of APC (Table 2). Adding APC to neurothrombectomy should strengthen the biological relationship between recanalization and outcome.

**Table 2:** **Challenges to the “recanalization hypothesis” and rationale for adjunctive APC**.

Recanalization problem	APC solution
No reflow phenomenon due to rethrombosis, migration of emboli, secondary thrombosis of downstream arteries, or microcirculatory occlusion	Antithrombotic activity
Reperfusion injury, hemorrhagic transformation, or cerebral edema,	Vasculoprotective effects on BBB integrity
Neurotoxicity	Neuroprotection
Recanalization occurs too late	Neurogenesis and Angiogenesis

*The potential explanations for poor clinical outcome despite reopening of occluded vessels and the beneficial properties of APC that redress each of these pitfalls are summarized*.

Firstly, recanalization of upstream large arteries is not always tantamount to tissue reperfusion distal to the occlusion. Rethrombosis, migration of emboli, secondary thrombosis of downstream arteries, or microcirculatory occlusion may produce a no-reflow phenomenon despite proximal vessel opening (Bai and Lyden, 2015). The inherent antithrombotic activity of APC might mitigate the deleterious clotting that underlies no-reflow. Conversely, excessive anticoagulation might promote intracerebral bleeding. APC variants such as 3K3A-APC, which have reduced anticoagulant activity compared with wild type, might represent a favorable compromise between these prothrombotic and anticoagulant forces, but this remains to be proven in human subjects.

Secondly, restoration of flow to ischemic brain tissue may risk reperfusion injury, hemorrhagic transformation, or cerebral edema, which could also counteract the theoretical benefit of recanalization. By repairing the integrity of the damaged BBB within the ischemic tissue, adjunctive APC confers vasculoprotective benefits.

Next, some recanalization therapies such as tPA have intrinsic neuorotoxicity through induction of caspases and other proapoptotic pathways, as well as or through breakdown of the BBB leading to the toxic accumulation of serum proteins that effect secondary neuronal injury (del Zoppo, 1998; Liu et al., 2004; Zlokovic and Griffin, 2011). The neuroprotective actions of APC might overcome this damage.

Lastly, recanalization may occur too late to benefit ischemic tissue that has already progressed to infarction. The neurogenic and angiogenic properties of APC, confirmed in both *in vitro* and *in vivo* models, might contribute to functional recovery and improved clinical outcome in such scenarios.

## Current Protocols and Future Directions

The “Safety Evaluation of 3K3A-APC in Ischemic Stroke (RHAPSODY)” trial (NCT02222714, NN104) is a multicenter, prospective, double-blinded, dose-escalation Phase 2 RCT. It intends to assess the safety, pharmacokinetics and efficacy of 3K3A-APC following treatment with tPA, mechanical neurothrombectomy, or both (for subjects undergoing neurothrombectomy, onset time to arterial puncture must be <6 h). Four different doses of 3K3A-APC are being tested to establish the maximum tolerated dose. Eligibility criteria include age 18–90 and NIHSS ≥5. The trial started in October 2014 and will enroll up to 100 subjects.

Prior studies in sepsis have shown that low-dose continuous infusion of APC is not optimally suited to harness its cell signaling actions and that bolus dosing more effectively promotes the receptor activation that leads to altered gene expression profiles and the salutary effects of BBB stabilization and of anti-apoptic and anti-inflammatory activities (Griffin et al., 2015). For this reason, the RHAPSODY protocol employs a regimen of intravenous APC bolus doses every 12 h, up to a total of five doses. The previous Phase 1 safety study in normal subjects confirmed that high dose bolus regimens using 3K3A-APC are safe (Lyden et al., 2013).

In the future, consideration can be given to direct intra-arterial administration of APC at the time of neurothrombectomy. When given through a microcatheter that has been navigated past the occlusion, intra-arterial injection can achieve better and more reliable delivery to the affected territory, even if the proximal vessel subsequently re-occludes and there is no recanalization. The intra-arterial delivery of APC after neurothrombectomy exemplifies the concept of endovascular restorative neurosurgery (ERN) that we previously advanced (Amar et al., 2003). Theoretic advantages of ERN with intra-arterial drug delivery include the possibility of widespread distribution, the capability to deliver large volumes and doses to target tissue relative to IV infusion, the ability to bypass the occlusive lesion and access territory not perfused by collateral flow, limited perturbation of neural tissue, and the feasibility of repeated administration (Amar et al., 2003). The relatively high dose of drugs like APC that can be delivered intra-arterially via ERN strategies support the biological rationale for this approach (Amar et al., 2003).

## Conclusion

An approach that couples mechanical neurothrombectomy with adjunctive delivery of a multiple action—multiple target drug offers many advantages over conventional treatment of AIS. The anti-inflammatory, anti-apoptotic, neuroprotective, and neuroregenerative properties of APC make this agent a compelling candidate for this strategy. While current protocols employ intravenous delivery of this agent, future studies of intra-arterial delivery are warranted.

## Funding

The authors want to acknowledge the NIH grants 9R01NS090904–16 to BVZ and RO1HL052246 and PO1 HL031950 to JHG for support for development of activated protein C analogs and mimetic peptides for stroke.

## Author Contributions

All authors (AA, JH, and BZ) fulfill the following criteria:

- Substantial contributions to the conception or design of the work; or the acquisition, analysis, or interpretation of data for the work;
- Drafting the work or revising it critically for important intellectual content;
- Final approval of the version to be published; and
- Agreement to be accountable for all aspects of the work in ensuring that questions related to the accuracy or integrity of any part of the work are appropriately investigated and resolved.

## Conflict of Interest Statement

Dr. Arun Paul Amar received time-based, market-value compensation for serving on the clinical events committees that adjudicated adverse outcomes in the Multi MERCI, SWIFT, and SWIFT PRIME trials. Dr. John H. Griffin is a consultant for ZZ Biotech LLC and inventor for some uses of 3K3A-APC. Dr. Berislav V. Zlokovic is a founder and the Chief Scientific Officer of ZZ Biotech LLC, a biotechnology company with a mission to develop APC and its functional mutants for the treatment of stroke and other neurological disorders.

## Abbreviations

APC: Activated protein C
AIS: Acute ischemic stroke
BBB: Blood brain barrier
CNS: Central nervous system
ERN: Endovascular restorative neurosurgery
ESCAPE: Endovascular Treatment for Small Core and Anterior Circulation Proximal Occlusion with Emphasis on Minimizing CT to Recanalization Times
EXTEND IA: Extending the Time for Thrombolysis in Emergency Neurological Deficits—Intra-Arterial
FAST MAG: Field Administration
IMS: Interventional Management of Stroke
IAT: Intra-arterial therapy
IV: Intravenous
IVT: Intravenous thrombolysis
LVO: Large vessel occlusion
MMP: Matrix metalloproteinase
MERCI: Mechanical Embolus Removal in Cerebral Ischemia
MR RESCUE: Mechanical Retrieval and Recanalization of Stroke Clots Using Embolectomy
MCAO: Middle cerebral artery occlusion
mRS: Modified Rankin score
Multi-MERCI: Multi Mechanical Embolus Removal in Cerebral Ischemia
MR CLEAN: Multicenter Randomized Clinical Trial of Endovascular Treatment for Acute Ischemic Stroke in the Netherlands
NINDS: National Institute of Neurological Disorders and Stroke
NIHSS: National Institutes of Health Stroke Scale
NR: Not reported
NF-κB: Nuclear factor kappa B
PROACT: Prolyse in Acute Cerebral Thromboembolism
PAR: Protease activated receptor
RCT: Randomized clinical trial
rpro-UK: Recombinant pro-urokinase
SWIFT: Solitaire With the Intention For Thrombectomy
SWIFT PRIME: Solitaire With the Intention For Thrombectomy as Primary Endovascular Treatment
STAR: Solitaire FR Thrombectomy for Acute Revascularization
SARIS: Stent assisted recanalization in acute ischemic stroke
STAIR: Stroke Therapy Academic Industry Roundtable
sICH: Symptomatic intracerebral hemorrhage
TICI: Thombolysis in cerebral infarction
TIMI: Thombolysis in myocardial infarction
tPA: Tissue plasminogen activator
FDA: United States Food and Drug Administration.
